# Supplementary material for: The causal relationship between the human gut microbiota and pyogenic arthritis: a Mendelian randomization study
Source: Front Cell Infect Microbiol. 2024 Nov 26;14:1452480. doi: 10.3389/fcimb.2024.1452480 (PMC11629706; doi:10.3389/fcimb.2024.1452480)
Supplement: Supplementary file 1 [file DataSheet1.zip › mendelian test/ebi-a-GCST90027586.csv_forest.pdf]

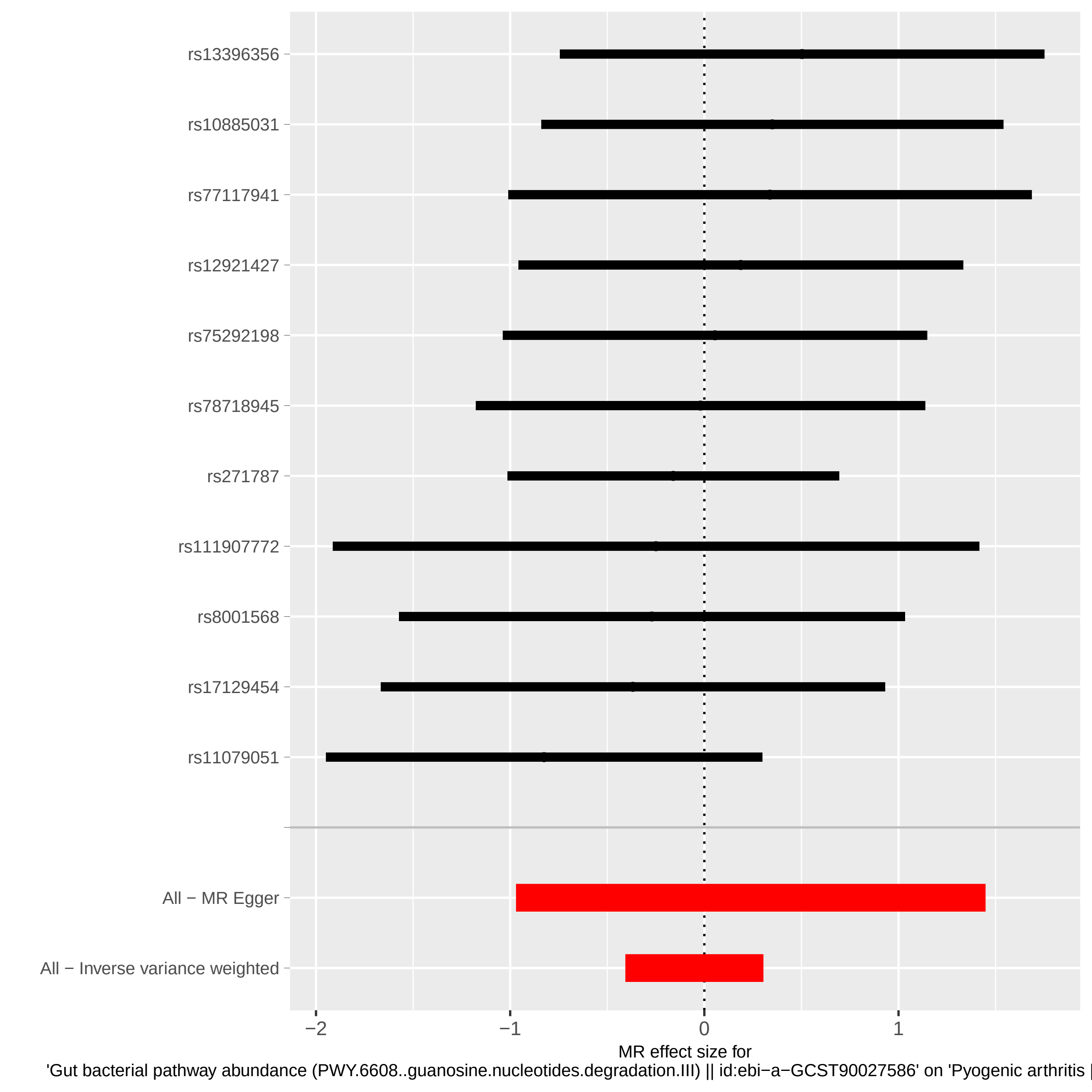

rs13396356
rs10885031
rs77117941
rs12921427
rs75292198
rs78718945
rs271787
rs111907772
rs8001568
rs17129454
rs11079051
All − MR Egger
All − Inverse variance weighted
−2
−1
0
1
MR effect size for
'Gut bacterial pathway abundance (PWY.6608..guanosine.nucleotides.degradation.III) || id:ebi−a−GCST90027586' on 'Pyogenic arthritis |
